# Family-Based SPL Model Checking Using Parity Games with Variability

**DOI:** 10.1007/978-3-030-45234-6_12

**Published:** 2020-03-13

**Authors:** Maurice H. ter Beek, Sjef van Loo, Erik P. de Vink, Tim A. C. Willemse

**Affiliations:** 8 University of Paderborn, Paderborn, Germany; 9 ICREA, Open University of Catalonia, Barcelona, Spain; 10 ISTI–CNR, Pisa, Italy; 11 TU Eindhoven, Eindhoven, The Netherlands

## Abstract

Family-based SPL model checking concerns the simultaneous verification of multiple product models, aiming to improve on enumerative product-based verification, by capitalising on the common features and behaviour of products in a software product line (SPL), typically modelled as a featured transition system (FTS). We propose efficient family-based SPL model checking of modal $\mu$-calculus formulae on FTSs based on variability parity games, which extend parity games with conditional edges labelled with feature configurations, by reducing the SPL model checking problem for the modal $\mu$-calculus on FTSs to the variability parity game solving problem, based on an encoding of FTSs as variability parity games. We validate our contribution by experiments on SPL benchmark models, which demonstrate that a novel family-based algorithm to collectively solve variability parity games, using symbolic representations of the configuration sets, outperforms the product-based method of solving the standard parity games obtained by projection with classical algorithms.

### 1 Introduction

Software product line engineering (SPLE) is a software engineering method for cost-effective and time-efficient development of a family of software-intensive configurable systems, according to which individual products (system variants) can be distinguished by the features they provide, where a feature is typically understood as some user-aware (difference in) functionality [1,2]. The intrinsic variability of SPLs challenges formal methods and analysis tools, because the number of possible products may be exponential in the number of features and each product may moreover exhibit a large behavioural state space.

The SPL model checking problem, first recognised in the seminal paper [3], generalises the classical model checking problem in the following way: given a formula, determine for each product whether it satisfies the formula (and, ideally, provide a counterexample for each product that does not satisfy the formula). A straightforward way to solve this problem is to provide a model for each product and apply classical model checking. This enumerative, product-based method has several drawbacks. Most importantly, the state-space explosion problem –typical of model checking– is amplified with the number of products, while products of a product line usually have a large amount of features and behaviour in common.

Therefore, Classen et al. have extended labelled transition systems (LTSs) with features to concisely describe and analyse the combined behaviour of a family of models [3–5]. Concretely, transitions in the resulting featured transition systems (FTSs) are labelled with actions and feature expressions. Given a product, a transition can be executed if the product fulfills the feature expression. Hence, an FTS incorporates all eligible product behaviour, and each individual product's behaviour can be obtained as an LTS. Moreover, FTSs cater for the simultaneous verification of multiple products, known as family-based analysis [6].

Properties of behavioural models for SPLs such as FTSs can be verified with dedicated SPL model checkers like SNIP [7], ProVeLines [8], VMC [9], ProFeat [10,11], or QFLan [12,13], or with classical model checkers like NuSMV [14, 15], SPIN [16], Maude [17], or mCRL2 [18, 19]. The advantage of using established off-the-shelf model checkers for SPL analysis is obvious: it lifts the burden of maintaining dedicated model checkers in favour of highly optimised tools with a broad user base. In [19], it was shown how to perform family-based SPL model checking with mCRL2 [20, 21] of properties of FTSs expressed in a feature-oriented variant of the modal $\mu$-calculus to deal with transitions labelled with feature expressions [22]. However, this approach is based on a decision procedure for the binary partitioning of the product space into products that do and those that do not satisfy a given formula, and it is underlined that computing suitable partitionings for the conducted experiments is a largely manual activity.

In this paper, we present efficient family-based SPL model checking of modal $\mu$-calculus formulae on FTSs based on parity games with variability. Years after its introduction [3, 14], family-based model checking of SPLs or program families is still a popular topic [10, 16, 19, 23–26], including a few game-theoretic approaches based on solving (3-valued) model checking games on featured symbolic automata and on modal transition systems. A parity game is a 2-player turn-based graph game. It is well known that the model checking problem for modal $\mu$-calculus formulae on LTSs is equivalent to parity game solving, for which Zielonka defined a recursive algorithm that performs well in practice [27–29].

Here we introduce variability parity games as a generalisation of parity games with conditional edges labelled with feature configurations. We then show how the SPL model checking problem for modal $\mu$-calculus formulae on FTSs can be reduced to the variability parity game solving problem based on an encoding of FTSs as variability parity games. Finally, we show the results of implementing two different methods, product-based and family-based, to solve variability parity games and of experimenting with them on two well-known SPL case studies, the minepump and the elevator. The product-based method simply projects a variability parity game to the different configurations and independently solves all resulting parity games with existing algorithms. The family-based method, instead, is based on a novel algorithm to collectively solve variability parity games, using symbolic representations of sets of configurations. The experiments clearly show that the family-based method outperforms the product-based method.

*Outline.* After defining some preliminary notions in Section 2, we introduce SPL model checking in Section 3. In Section 4, we introduce variability parity games and show how they can be used to solve the SPL model checking problem.

In Section 5, we present a family-based, collective strategy for recursively solving variability parity games, which we experiment with on two SPL case studies in Section 6. Section 7 concludes the paper and provides directions for future work. Relevant related work other than the above is mentioned throughout the paper.

### 2 Preliminaries

We give a brief overview of *labelled transition systems* and the *modal $\mu$-calculus.*

**Definition 1.** *A labelled transition system or LTS $L$ over a non-empty set of actions $\mathcal{A}ct$ is a triple $L = (S, \rightarrow, s_0)$, where $S$ is the set of states with $s_0 \in S$ and $\rightarrow \subseteq S \times \mathcal{A}ct \times S$ is the transition relation.*

The modal $\mu$-calculus is an expressive logic, subsuming LTL and CTL, for reasoning about the behaviours of LTSs, among others.

**Definition 2.** *Formulae in the modal $\mu$-calculus are given by the following (minimal) grammar.*

$$\phi ::= \mathsf{true} \mid \mathsf{false} \mid X \mid \phi \wedge \phi \mid \phi \vee \phi \mid \langle a \rangle \phi \mid [a]\phi \mid \mu X.\phi \mid \nu X.\phi$$

*where $a \in \mathcal{A}ct$ is an action and $X \in \mathcal{X}$ is some propositional variable taken from a sufficiently large set of variables $\mathcal{X}$.*

Next to the Boolean constants and the propositional connectives, the modal $\mu$-calculus contains the existential diamond operator $\langle\ \rangle$ and its dual universal box operator $[\ ]$ of modal logic as well as the least and greatest fixed point operators $\mu$ and $\nu$ that provide recursion used for 'finite' and 'infinite' looping, respectively.

Given a formula $\phi$, an occurrence of a variable $X$ in $\phi$ is said to be *bound* iff this occurrence is within a formula $\psi$, where $\mu X.\psi$ or $\nu X.\psi$ is a subformula of $\phi$; an occurrence of a variable is *free* otherwise. A formula $\phi$ is *closed* iff all variables occurring in $\phi$ are bound; here we only consider closed formulae. For simplicity, we assume that the formulae that we consider are *well-named*, *i.e.*, formulae do not contain two fixed point subformulae binding the same variable.

Given an LTS, the semantics of a $\mu$-calculus formula is the set of states of the LTS that satisfy the formula. Since we focus on games in this paper, we introduce two auxiliary concepts, *viz.* the *Fischer-Ladner closure* of a formula and the *alternation depth* of a formula. The Fischer-Ladner closure $FL(\phi)$ of a formula $\phi$ is the smallest set of formulae satisfying

- $\phi \in FL(\phi)$;
- if $\phi_1 \wedge \phi_2 \in FL(\phi)$ or $\phi_1 \vee \phi_2 \in FL(\phi)$ then $\phi_1, \phi_2 \in FL(\phi)$;
- if $\langle a \rangle \phi_1 \in FL(\phi)$ or $[a]\phi_1 \in FL(\phi)$ then $\phi_1 \in FL(\phi)$;
- if $\sigma X.\phi_1 \in FL(\phi)$ then $\phi_1[X := \sigma X.\phi_1] \in FL(\phi)$.

Note that for a closed formula $\phi$, the set $FL(\phi)$ contains no variables.

The complexity of a $\mu$-calculus formula is given by its *alternation depth*; the larger the alternation depth, the harder the formula is to solve (and, incidentally, also to understand). The alternation depth of a formula $\phi$ is defined as the largest alternation depth of the bound propositional variables in $\phi$, defined as follows.

**Definition 3.** *The* dependency order *on bound variables of a formula $\phi$ is the smallest partial order $\leq_\phi$ satisfying $X \leq_\phi Y$ if $X$ occurs free in $\sigma Y.\psi$. The* alternation depth *of a $\mu$-variable $X$ in $\phi$, denoted $AD_\phi(X)$, is the maximal length of a chain $X_1 \leq_\phi \cdots \leq_\phi X_n$, where $X_1 = X$, variables $X_1, X_3, \ldots$ are $\mu$-variables and $X_2, X_4, \ldots$ are $\nu$-variables. Analogously for the alternation depth of a $\nu$-variable.*

**Definition 4.** *A parity game is a tuple $G = (V, E, p, (V_0, V_1))$ where*

- *$V$ is a finite set of vertices, partitioned into a set $V_0$ of vertices owned by player 0 and a set $V_1$ of vertices owned by player 1;*
- *$E \subseteq V \times V$ is the edge relation;*
- *$p : V \to \mathbb{N}$ is the priority function.*

We depict parity games as graphs in which diamond-shaped vertices represent vertices owned by player 0 and box-shaped vertices represent vertices owned by player 1. Edges are annotated with configurations while priorities are typically written inside vertices.

We write $v \to w$ instead of $(v, w) \in E$ and let $\alpha$ range over the set of players, *i.e.* $\alpha \in \{0, 1\}$. For a given vertex $v$, we write $vE$ to denote the set $\{w \in V \mid v \to w\}$ of successors of $v$. Likewise, $Ev$ denotes the set $\{w \in V \mid w \to v\}$ of predecessors of $v$. A sequence of vertices $v_1 \cdots v_n$ is a *path* if for all $1 \leqslant m < n$ we have $v_{m+1} \in v_m E$. Infinite paths are defined in a similar way. We write $\pi_n$ to denote the $n$-th vertex in a path $\pi$ and $\pi^{\leqslant n}$ to indicate the prefix $\pi_1 \cdots \pi_n$ of $\pi$.

A play, starting in a vertex $v \in V$, starts by placing a token on that vertex. Players then move the token according to a single simple rule: if a token is on a vertex $u \in V_\alpha$ and $uE \neq \emptyset$, player $\alpha$ pushes it to some successor vertex $w \in uE$. The finite and infinite paths thus constructed are referred to as *plays*. For an infinite play, and the infinite sequence of priorities it induces, the *parity* of the highest priority that occurs infinitely often on that play defines its *winner*: player 0 wins if this priority is even; player 1 wins otherwise. A finite play is won by the player that does *not* own the vertex on which the token is stuck.

The moves of players 0 and 1 are determined by their respective *strategies*. Informally, a strategy for a player $\alpha$ determines, for a vertex $\pi_i \in V_\alpha$ the next vertex $\pi_{i+1}$ that will be visited if a token is on $\pi_i$, provided $\pi_i$ has successors. In general, a strategy is a partial function $\sigma : V^* V_\alpha \to V$ which, for a given history of vertices of the locations of the token and a vertex on which the token currently resides, determines the next vertex by selecting an edge to that vertex. A finite or infinite path $\pi$ *conforms to* a given strategy $\sigma$ if for all prefixes $\pi^{\leqslant i}$ for which $\sigma$ is defined, we have $\pi_{i+1} = \sigma(\pi^{\leqslant i})$.

A strategy $\sigma$ for player $\alpha$ is *winning* from a vertex $v$ iff $\alpha$ is the winner of every play starting in $v$ that conforms to $\sigma$. Parity games are known to be *positionally determined* [30]. This means that a vertex is won by player $\alpha$ iff $\alpha$ has a winning strategy that does not depend on the history of vertices visited by the token. Such strategies can be represented by partial functions $\sigma : V_\alpha \to V$. Note that every vertex in a parity game is won by one of the two players.

Closed modal $\mu$-calculus formulae can be interpreted by associating a game semantics to these formulae. The definition we provide below is adopted from [30].

**Table 1.** The game semantics for a closed modal $\mu$-calculus formula $\phi$: vertex $v$ (1st column), its owner $\alpha$ (2nd column), its successors (if any) $w \in vE$ (3rd column), and priority $p(v)$ (4th column). Vertices of the form $(s, \langle a \rangle \psi)$ and $(s, [a]\psi)$ have no successors when $s$ has no $a$-successors.

| Vertex | Owner | Successor(s) | Priority |
|---|---|---|---|
| $(s, \mathsf{true})$ | 1 | | 0 |
| $(s, \mathsf{false})$ | 0 | | 0 |
| $(s, \psi_1 \wedge \psi_2)$ | 1 | $(s, \psi_1)$ and $(s, \psi_2)$ | 0 |
| $(s, \psi_1 \vee \psi_2)$ | 0 | $(s, \psi_1)$ and $(s, \psi_2)$ | 0 |
| $(s, [a]\psi)$ | 1 | $(t, \psi)$ for every $s \xrightarrow{a} t$ | 0 |
| $(s, \langle a \rangle \psi)$ | 0 | $(t, \psi)$ for every $s \xrightarrow{a} t$ | 0 |
| $(s, \nu X.\psi)$ | 1 | $(s, \psi[X := \nu X.\psi])$ | $2\lfloor AD_\phi(X)/2 \rfloor$ |
| $(s, \mu X.\psi)$ | 1 | $(s, \psi[X := \mu X.\psi])$ | $2\lfloor AD_\phi(X)/2 \rfloor + 1$ |

**Definition 5.** *Let $L = (S, \rightarrow, s_0)$ be an LTS and $\phi$ be a closed modal $\mu$-calculus formula. A state $s \in S$ satisfies formula $\phi$, denoted by $L, s \models \phi$, iff vertex $(s, \phi)$ is won by player 0 in the game $G_{L,\phi} = (V, E, p, (V_0, V_1))$, where $V = S \times FL(\phi)$, and the sets $E$, $V_0$, and $V_1$ and priority function $p$ are given by Table 1.*

If the context is such that no confusion can arise, we write $s \models \phi$ for $L, s \models \phi$.

For a more in-depth treatment of the modal $\mu$-calculus, we refer to [30]. Here, we finish by illustrating the game semantics on a small example, drawing inspiration from an example in [19].

*Example 1.* Consider the LTS $L$ depicted in the bottom-left corner of Fig. 1, modelling a coffee machine that after inserting one or two units of some currency (indicated by action *ins*) can dispense a standard regular coffee (indicated by action *std*) or an extra large coffee (indicted by action *xxl*), respectively.

The LTL-type formula $\phi$, depicted in the top-left corner of Fig. 1, asserts that on all infinite runs of the coffee machine, it infinitely often dispenses a regular coffee. (Note, nothing is required to hold on finite runs.) The parity game that can answer whether $s_0 \models \phi$ holds is depicted on the right in Fig. 1. Each node is annotated with a pair consisting of a state of the LTS and a (sub)formula of $\phi$. Note that the references to $\phi_1, \phi_2$, and $\phi_3$ are meant as an indication and not to be interpreted exactly, since they lack the substitution that needs to be carried out. We remark that the parity game is *solitair*: only one player can make decisions. Vertex $(s_0, \phi)$ is won by player 1 by enforcing a 1-dominated infinite play, bypassing the vertex with priority 2 on the loop. Consequently, $s_0 \not\models \phi$. □

### 3 Software Product Lines Model Checking

Software products with variability can be modelled effectively using so-called *featured transition systems* or FTSs [3]. Fix a finite non-empty set $\mathcal{F}$ of features, with $\mathtt{f}$ as typical element. Let $\mathbb{B}[\mathcal{F}]$ denote the set of Boolean expressions over $\mathcal{F}$. Elements $\chi$ and $\gamma$ of $\mathbb{B}[\mathcal{F}]$ are referred to as feature expressions. A product $\mathtt{P}$ is a set of features, $\mathcal{P}$ denotes the set of products, thus $\mathcal{P} \subseteq \mathbf{2}^{\mathcal{F}}$.

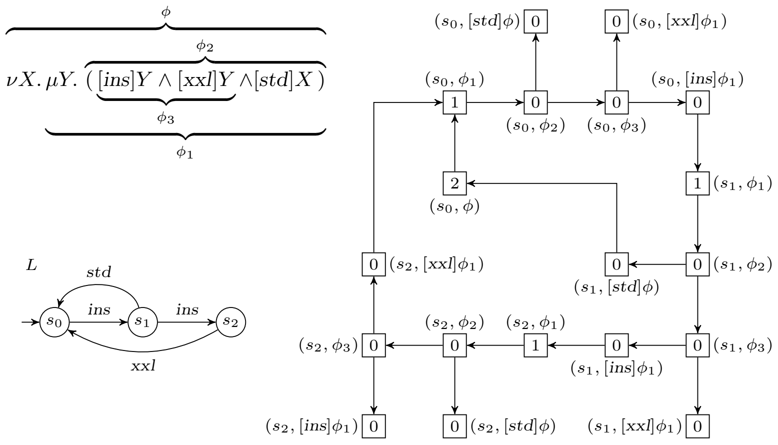

**Fig. 1.** Parity game encoding the model checking problem $s_0 \models \phi$

A feature expression $\gamma$, as Boolean expression over $\mathcal{F}$, can be interpreted as a set of products $P_\gamma$, *viz.* all products $\mathtt{P}$ for which the induced truth assignment ($\mathsf{true}$ for $\mathtt{f} \in \mathtt{P}$, $\mathsf{false}$ for $\mathtt{f} \notin \mathtt{P}$) validates $\gamma$. Reversely, for each family $P \subseteq \mathcal{P}$ we fix a feature expression $\gamma_P$ to represent it. The constant $\top$ denotes the feature expression that is always true. We now recall FTSs from [4] as a model for software product lines, using the notation of [19, 22].

**Definition 6.** *An FTS $F$ over $\mathcal{A}ct$ and $\mathcal{F}$ is a triple $F = (S, \theta, s_0)$, where $S$ is the set of states with $s_0 \in S$ and $\theta : S \times \mathcal{A}ct \times S \to \mathbb{B}[\mathcal{F}]$ is the transition constraint function.*

For states $s, t \in S$, we write $s \xrightarrow{a|\gamma}_F t$ if $\theta(s, a, t) = \gamma$ and $\gamma \neq \bot$. The projection of $F$ onto a product $\mathtt{P} \in \mathcal{P}$ is the LTS $F|\mathtt{P} = (S, \to_{F|\mathtt{P}}, s_0)$ over $\mathcal{A}ct$ with $s \xrightarrow{a}_{F|\mathtt{P}} t$ iff $\mathtt{P} \in P_\gamma$ for a transition $s \xrightarrow{a|\gamma}_F t$ of $F$.

*Example 2.* Assume that the coffee machine from Example 1 is to model a family of coffee machines for different countries, depending on whether a coffee machine accepts the insertion of dollars or euros, or both. Let $P$ be a product line of coffee machines, with the independent features \$ and €, representing the presence of a coin slot accepting dollars or euros, respectively, leading to a set of four products: $\{\varnothing, \{\$\}, \{€\}, \{\$, €\}\}$. The FTS $F$ below models the family behaviour of $P$.

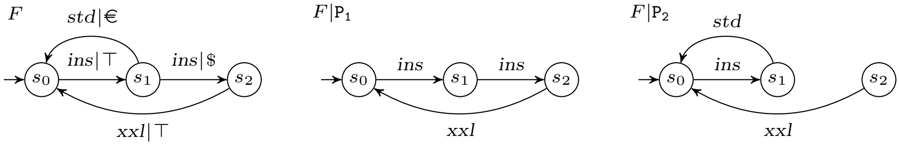

The idea is that extra large coffee is exclusively available for 2 dollars, whereas 1 euro or dollar suffices for a standard regular coffee. The behaviour of products $\mathtt{P}_1 = \{\$\}$ and $\mathtt{P}_2 = \{€\}$ is modelled by the LTSs $F|\mathtt{P}_1$ and $F|\mathtt{P}_2$ depicted above.

Note that coffee machine $F|\mathtt{P}_1$ accepting only dollars lacks the transition from $s_1$ to $s_0$ requiring feature €, while coffee machine $F|\mathtt{P}_2$ accepting only euros lacks the one from $s_1$ to $s_2$ requiring feature \$. The behaviour of product $\mathtt{P}_3 = \{\$, €\}$ is modelled by the LTS $L = F|\{\$, €\}$ depicted in Fig. 1. Finally, the product without any features is not depicted, but it deadlocks at state $s_1$. □

**Definition 7.** *The* SPL model checking problem *is to compute, for a given FTS* $F = (S, \theta, s_0)$ *and closed modal μ-calculus formula* $\phi$*, the largest subsets* $P^+$ *and* $P^-$ *of* $\mathcal{P}$ *such that* $F|\mathtt{P}, s_0 \models \phi$ *for all* $\mathtt{P} \in P^+$ *and* $F|\mathtt{P}, s_0 \not\models \phi$ *for all* $\mathtt{P} \in P^-$*.*

Sets $P^+$ and $P^-$ partition $\mathcal{P}$: a formula either does or does not hold in a state.

*Example 3.* It is not difficult to see that the formula $\phi$ from Example 1 does not hold for all products. In fact, $P^+ = \{\varnothing, \{€\}\}$ and $P^- = \{\{\$\}, \{\$, €\}\}$. For products with feature \$, there is an infinite run that avoids action *std* altogether, whereas for products not containing feature \$, either all runs are finite, or all infinite runs contain an infinite number of *std* actions. □

### 4 Variability Parity Games and SPL Model Checking

In practice, the model checking problem for LTSs, yielding a *yes/no* answer, can efficiently be decided using parity game solving algorithms [27,30]. The SPL model checking problem can be solved in a similar fashion by constructing parity games associated with the formula and with each individual product separately. Such an approach, however, does not take full advantage of the efficient, compact representation of the variation points in the individual product LTSs represented by an FTS. The *variability parity games* we introduce in Section 4.1, exploit constructs similar to those in FTSs to compactly encode variation points in the parity games they represent. We show in Section 4.2 that the SPL model checking problem can be solved by solving such variability parity games.

#### 4.1 Variability Parity Games

A variability parity game is a generalisation of a parity game. It is a two-player game, again played by players *odd*, denoted by 1, and *even*, denoted by 0, on a finite directed graph. Contrary to parity games, an edge in a variability parity game is associated with a set of *configurations*.

**Definition 8 (Variability Parity Game).** *A variability parity game* $\mathcal{G}$ *is a sextuple* $\mathcal{G} = (V, E, \mathfrak{C}, p, \theta, (V_0, V_1))$*, where*

- $V$ *is a finite set of vertices, partitioned into sets* $V_0$ *and* $V_1$ *of vertices owned by player* 0 *and player* 1*, respectively;*
- $E \subseteq V \times V$ *is the edge relation;*
- $\mathfrak{C}$ *is a finite set of configurations;*
- $p : V \to \mathbb{N}$ *is the priority function that assigns priorities to vertices;*
- $\theta : E \to 2^{\mathfrak{C}} \setminus \{\emptyset\}$ *is the configuration mapping.*

In line with our depiction of parity games, we visualise variability parity games as graphs with diamond-shaped and box-shaped vertices, and directed edges connecting vertices. Moreover, edges are annotated with configurations. A variability parity game $\mathcal{G} = (V, E, \mathfrak{C}, p, \theta, (V_0, V_1))$ is called *total* if, for all $u \in V$, it holds that $\bigcup\{\, \theta(u,v) \mid v \in V,\ (u,v) \in E \,\} = \mathfrak{C}$.

As before, we write $v \rightarrow w$ for $(v, w) \in E$, and we use $\alpha$ to range over $\{0, 1\}$. We use $v \xrightarrow{c} w$ to denote $v \rightarrow w$ and $c \in \theta(v, w)$ and say that the edge between $v$ and $w$ is *compatible* with $c$. The notions of a finite and infinite path from parity games carry over to variability parity games, and we use similar notation to denote the prefixes of a path and the vertices along a path. A finite path $v_1 \cdots v_n$ is *admitted* for a configuration $c \in \mathfrak{C}$ iff for all $m < n$, $c \in \theta(v_m, v_{m+1})$. In a similar vein, an infinite path can be said to be admitted for a given configuration.

A play starts by placing a *configured* token $c \in \mathfrak{C}$ on vertex $v \in V$. The players move configured token $c$ in the game according to the following rule: if token $c \in \mathfrak{C}$ is on some vertex $v \in V_\alpha$, player $\alpha$ pushes $c$, if possible, to some adjacent vertex $w$ along an edge compatible with $c$, *i.e.* $c \in \theta(v, w)$. The finite and infinite paths thus constructed are admitted by $c$, and are again referred to as *plays*; the conditions for players 0 and 1 for winning such plays are identical to those for parity games.

For a configuration $c \in \mathfrak{C}$, a strategy is a partial function $\sigma_c : V^* V_\alpha \rightarrow V$ which, when defined for $\pi^{\leqslant i}$, yields a vertex $\pi_{i+1}$ that is reachable from $\pi_i$ via an edge that is compatible with $c$. A path $\pi$, admitted by configuration $c$, *conforms to* a given strategy $\sigma_c$ iff for all prefixes $\pi^{\leqslant i}$ for which $\sigma$ is defined, we have $\pi_{i+1} = \sigma_c(\pi^{\leqslant i})$. Strategy $\sigma_c$ for player $\alpha$ and configuration $c$ is *winning* from a vertex $v$ iff $\alpha$ is the winner of every play starting in $v$ that conforms to $\sigma_c$.

**Definition 9.** *The* variability parity game solving problem *for a vertex $v$ is the problem of computing the largest set of configurations $C_0, C_1 \subseteq \mathfrak{C}$ such that:*

- *player 0 has a winning strategy for $v$ for each $c \in C_0$;*
- *player 1 has a winning strategy for $v$ for each $c \in C_1$.*

For a given variability parity game $\mathcal{G}$ and a configuration $c \in \mathfrak{C}$, we define the projection of $\mathcal{G}$ onto $c$, denoted $\mathcal{G}|c$ as the parity game obtained by retaining only those edges from $\mathcal{G}$ that are compatible with $c$. We note that it follows rather immediately that variability parity games are also *positionally determined*: player 0 (player 1, respectively) has a winning strategy $\sigma_c$ for vertex $v$ for configuration $c$ iff she has a winning strategy for $v$ in the projection of the variability parity game onto configuration $c$. Since parity games are positionally determined, so are variability parity games. Consequently, the variability parity game solving problem asks for the computation of a partition of the set of configurations $\mathfrak{C}$.

#### 4.2 Solving SPL Model Checking Using Variability Parity Games

If we ignore the representation of the sets of configurations decorating the edges, a variability parity game is a compact representation of a set of parity games. The

**Table 2.** Transformation of the SPL model checking problem to the variability parity game solving problem. For a given vertex $v$ (1st column), its owner $\alpha$ (2nd column), successors $w \in vE$ (3rd column) and configuration mapping $\theta(v, w)$ (3rd column), and priority $p(v)$ (4th column) are given.

| Vertex | Owner | Successor(s) \| Configurations | Priority |
|---|---|---|---|
| $(s, \mathsf{true})$ | 1 | | 0 |
| $(s, \mathsf{false})$ | 0 | | 0 |
| $(s, \psi_1 \wedge \psi_2)$ | 1 | $(s, \psi_1) \mid \mathcal{P}$ and $(s, \psi_2) \mid \mathcal{P}$ | 0 |
| $(s, \psi_1 \vee \psi_2)$ | 0 | $(s, \psi_1) \mid \mathcal{P}$ and $(s, \psi_2) \mid \mathcal{P}$ | 0 |
| $(s, [a]\psi)$ | 1 | $(t, \psi) \mid P_\gamma$ for every $s \xrightarrow{a\mid\gamma}_F t$ | 0 |
| $(s, \langle a \rangle \psi)$ | 0 | $(t, \psi) \mid P_\gamma$ for every $s \xrightarrow{a\mid\gamma}_F t$ | 0 |
| $(s, \nu X.\psi)$ | 1 | $(s, \psi[X := \nu X.\psi]) \mid \mathcal{P}$ | $2\lfloor AD_\phi(X)/2 \rfloor$ |
| $(s, \mu X.\psi)$ | 1 | $(s, \psi[X := \mu X.\psi]) \mid \mathcal{P}$ | $2\lfloor AD_\phi(X)/2 \rfloor + 1$ |

next definition shows how to exploit these configurations to efficiently encode the SPL model checking problem as a variability parity game solving problem, based on the game-based semantics of the modal $\mu$-calculus we presented in Section 2.

**Definition 10.** *Let $F = (S, \theta_F, s_0)$ be an FTS, let $\mathcal{P}$ be the set of all products, and let $\phi$ be a closed modal $\mu$-calculus formula. The variability parity game $F_\phi = (V, E, \mathfrak{C}, p, \theta, (V_0, V_1))$ associated with $F$ and $\phi$, with $V = S \times FL(\phi)$ and $\mathfrak{C} = \mathcal{P}$, is defined by the rules given in Table 2.*

Note that the size of the graph underlying variability parity game $F_\phi$, measured in terms of $|V| + |E|$, is linear in the size of formula $\phi$ and the FTS $F$, measured in terms of $|S| + |\{(s, a, t) \in S \times \mathcal{A}ct \times S \mid \theta(s, a, t) \neq \bot\}|$. Hence, the structural information in an FTS is compactly reflected in the variability parity game which encodes the SPL model checking problem for the FTS. The correctness of the encoding is expressed by the Theorem 1.

**Theorem 1.** *For a given FTS $F$, a closed modal $\mu$-calculus formula $\phi$, and a product $\mathtt{P}$, we have $F|\mathtt{P}, s \models \phi$ iff player 0 wins the vertex $(s, \phi)$ for configuration $\mathtt{P}$ in the variability parity game $F_\phi$ associated to $F$ and $\phi$.*

*Proof (sketch).* Fix an FTS $F$ and a closed modal $\mu$-calculus formula $\phi$. Let $\mathtt{P}$ be a product. It is not hard to show that the parity game we obtain by encoding the model checking problem $F|\mathtt{P}, s \models \phi$ (cf. Definition 5) is isomorphic to the projection of $F_\phi$ onto $\mathtt{P}$, *viz.* $F_\phi|\mathtt{P}$. □

We revisit the SPL model checking problem of Example 3, illustrating the encoding of Definition 10. By abuse of notation, we write feature expressions instead of sets of configurations in variability parity games associated to SPL model checking problems.

*Example 4.* Consider the FTS $F$ of Example 2 and the modal $\mu$-calculus formula $\phi$ of Example 1, both for convenience repeated in Fig. 2. The variability parity game $F_\phi$ encoding the SPL model checking problem for $F$ and $\phi$ is depicted on the right in Fig. 2 (ignoring all dashed self loops for now). We omitted most state annotations to yield a more readable figure.

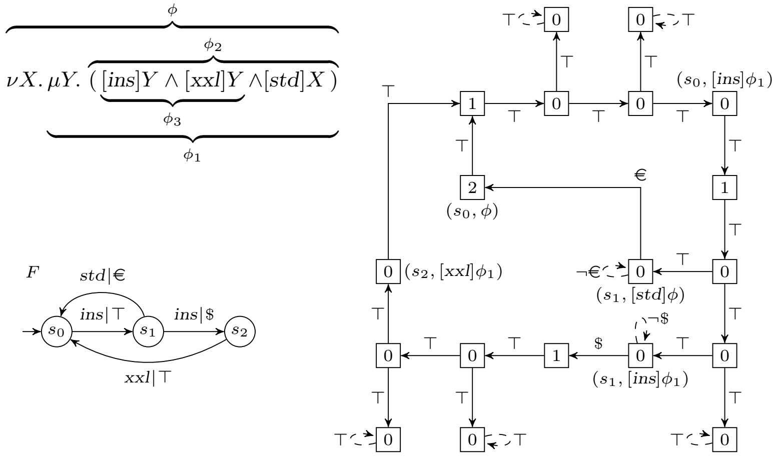

**Fig. 2.** Variability parity game encoding the SPL model checking problem for $F$ and $\phi$.

Observe that the graph structure of the variability parity game $F_\phi$ is the same as that of the parity game of Example 1 in Fig. 1. The construction leading to the variability parity game only differs in the construction of the parity game with respect to the edge annotations. Furthermore, note that vertex $(s_0, \phi)$ is won by player 0 for the set of configurations ¬\$, whereas player 1 wins the set of configurations \$: for configurations containing the feature \$, player 1 can essentially reuse the strategy of Example 1, avoiding the vertex with priority 2. For configurations not containing the feature \$, this option is not available, since the vertex $(s_1, [\mathit{ins}]\phi_1)$ is a sink. For products with feature € but not \$, the only infinite play infinitely often visits vertex $(s_0, \phi)$. For products without features € and \$ all plays starting in $(s_0, \phi)$ are finite. Hence, by Theorem 1, the solution to the SPL model checking problem is the pair (¬\$, \$), as expected. □

### 5 Recursively Solving Variability Parity Games

Given a variability parity game $\mathcal{G}$ and a vertex $v$ of $\mathcal{G}$, a straightforward way of solving the variability parity game problem for $v$ is by simply solving the standard parity game problem $\mathcal{G}|c$ for every $c \in \mathfrak{C}$. In doing so, however, we ignore that players can potentially use (parts of) a single strategy for possibly many different configurations. As opposed to the above solving strategy, to which we refer as the *individual solving strategy*, we investigate an alternative for variability parity games, called the *collective solving strategy*.

We provide an algorithm, Algorithm 1, for solving variability parity games inspired by the classical *recursive* algorithm for solving parity games [27]. The recursive algorithm is, despite its unappealing theoretical worst-case complexity, in practice one of the most effective algorithms for solving parity games [28,29]. It is a divide-and-conquer algorithm that relies on two building blocks, *viz.* the concept of a *subgame computation* and of an *attractor computation*. We generalise and adapt these concepts to the setting of variability parity games.

Fix a variability parity game $\mathcal{G} = (V, E, \mathfrak{C}, p, \theta, (V_0, V_1))$. For simplicity we assume that $\mathcal{G}$ is total. This is not a limitation; any variability parity game can be turned into a total one. The auxiliary notion of a *restriction* is a mapping $\varrho : V \to 2^{\mathfrak{C}}$ which, for a variability parity game $\mathcal{G}$, indicates which configurations are under consideration for a vertex. Given such a restriction $\varrho$, we say that a vertex $v$ for configuration $c \in \mathfrak{C}$ is won by player $\alpha$ in the game $\mathcal{G}$ restricted to $\varrho$ iff $c \in \varrho(v)$ and the winning strategy for $\alpha$ only passes through vertices $v'$ for which $c \in \varrho(v')$. We say that $\mathcal{G}$ is *total* with respect to $\varrho$ iff for all $v \in V$ and all $c \in \varrho(v)$, there is a vertex $w$ such that $w \in vE$ and $c \in \theta(v, w) \cap \varrho(w)$.

Let $U, U' : V \to 2^{\mathfrak{C}}$ be arbitrary mappings. The union of $U$ and $U'$, denoted $U \cup U'$, is defined point-wise, *i.e.* $(U \cup U')(v) = U(v) \cup U(v')$. We say that mapping $U$ is a *sub-mapping* of $\varrho$ iff for all $v \in V$ we have $U(v) \subseteq \varrho(v)$. The reduction of $\varrho$ with respect to a sub-mapping $U$, denoted $\varrho \backslash U$, is a new restriction defined as $(\varrho \backslash U)(v) = \varrho(v) \backslash U(v)$.

For a given sub-mapping $U : V \to 2^{\mathfrak{C}}$ of a restriction $\varrho$, the $\alpha$-*attractor* towards $U$ is a sub-mapping of $\varrho$ which assigns those configurations to a vertex for which player $\alpha$ can force the play to reach some vertex $v$ for which that configuration belongs to $U(v)$. Formally, we define $Attr_\alpha(U)$, in the context of $\varrho$ and $\mathcal{G}$, as $Attr_\alpha(U)(v) = \bigcup_{i \geq 0} Attr^i_\alpha(U)(v)$, where

$$
\begin{aligned}
&Attr^0_\alpha(U)(v) \quad = U(v)\\
&Attr^{i+1}_\alpha(U)(v) = Attr^i_\alpha(U)(v) \cup\\
&\qquad \{\, c \in \varrho(v) \mid v \in V_\alpha \wedge \exists w \in vE\colon c \in \theta(v,w) \cap \varrho(w) \cap Attr^i_\alpha(U)(w) \,\} \cup\\
&\qquad\qquad \{\, c \in \varrho(v) \mid v \in V_{\bar{\alpha}} \wedge \forall w \in vE\colon c \in (\mathfrak{C} \backslash (\theta(v,w) \cap \varrho(w))) \cup Attr^i_\alpha(U)(w) \,\}
\end{aligned}
$$

Thus, in case $v \in V_\alpha$ and $c \in \varrho(v)$, configuration $c$ is in $Attr^{i+1}_\alpha(U)(v)$ if for a move by player $\alpha$ to some vertex $w$ allowed for configuration $c$, the sub-attractor $Attr^i_\alpha(U)(w)$ can be reached. In case $v \in V_{\bar{\alpha}}$ and $c \in \varrho(v)$, configuration $c$ is in $Attr^{i+1}_\alpha(U)(v)$ if all moves for player $\bar{\alpha}$ are not allowed for configuration $c$ or lead to a vertex $w$ in the sub-attractor $Attr^i_\alpha(U)(w)$ for player $\alpha$ for $A$.

*Example 5.* Reconsider the variability parity game of Example 4. First, observe that it is not total. In this case, the variability parity game can be made total without changing the solution by taking into account also the dashed self loops.

Let $\varrho(v) = \mathfrak{C}$ and define $U(s_0, \phi) = \mathfrak{C}$ and $U(v) = \varnothing$ for all $v \neq (s_0, \phi)$. For vertex $(s_0, \phi)$ we have $Attr_0(U)(s_0, \phi) = \{\varnothing, \{\$\}, \{€\}, \{\$, €\}\}$. All vertices $v$ on the (single) path starting in $(s_0, [ins]\phi_1)$ and ending in $(s_1, [std]\phi)$ satisfy $Attr_0(U)(v') = \{\{\$, €\}\}$. The remaining vertices $v'$ satisfy $Attr_0(U)(v') = \varnothing$. Note that for no configuration the immediate predecessor of $(s_0, [ins]\phi_1)$ is attracted to $U$ because of the escape to the sink that player 1 can use. □

We have the following result, which can be proven by induction on $i$ following the definition of $Attr_\alpha(U)(v) = \bigcup_{i \geq 0} Attr^i_\alpha(U)(v)$.

**Algorithm 1** Recursive Algorithm for a fixed variability parity game $\mathcal{G} = (V, E, \mathfrak{C}, p, \theta, (V_0, V_1))$. Given a restriction $\varrho : V \to 2^{\mathfrak{C}}$, the algorithm returns a pair of functions $(W_0, W_1)$ where $W_0, W_1 : V \to 2^{\mathfrak{C}}$ denote, for each vertex, which set of configurations is won by player 0 (player 1, respectively).

**function** $\text{Solve}(\varrho)$<br>
    **if** $\varrho = \lambda v \in V.\, \emptyset$ **then**<br>
        $(W_0, W_1) \leftarrow (\lambda v \in V.\, \emptyset, \lambda v \in V.\, \emptyset)$<br>
    **else**<br>
        $m \leftarrow \max\{\, p(v) \mid v \in V \wedge \varrho(v) \neq \emptyset \,\}$<br>
        $\alpha \leftarrow m \mod 2$<br>
        $U \leftarrow \lambda v \in V.\, \{\varrho(v) \mid p(v) = m\}$<br>
        $A \leftarrow Attr_\alpha(U)$<br>
        $(W_0', W_1') = \text{Solve}(\varrho \backslash A)$<br>
        **if** $W_{\bar{\alpha}'} = \lambda v \in V.\, \emptyset$ **then**<br>
            $W_\alpha \leftarrow W_\alpha' \cup A$<br>
            $W_{\bar{\alpha}} \leftarrow W_{\bar{\alpha}}'$<br>
        **else**<br>
            $B \leftarrow Attr_{\bar{\alpha}}(W_{\bar{\alpha}}')$<br>
            $(W_0'', W_1'') = \text{Solve}(\varrho \backslash B)$<br>
            $W_\alpha \leftarrow W_\alpha''$<br>
            $W_{\bar{\alpha}} \leftarrow W_{\bar{\alpha}}'' \cup B$<br>
        **end if**<br>
    **end if**<br>
    **return** $(W_0, W_1)$<br>
**end function**

**Lemma 1.** *Let $\mathcal{G} = (V, E, \mathfrak{C}, p, \theta, (V_0, V_1))$ be a variability parity game, let $\varrho : V \to 2^{\mathfrak{C}}$ a restriction, and let $\alpha$ be an arbitrary player. Then for all sub-mappings $U$ of $\varrho$, also $Attr_\alpha(U)$ is a sub-mapping of $\varrho$.* □

Totality of a game is preserved for the complements of attractors of sub-mappings.

**Lemma 2.** *Let $\mathcal{G} = (V, E, \mathfrak{C}, p, \theta, (V_0, V_1))$ be a variability parity game and let $\varrho : V \to 2^{\mathfrak{C}}$ be a restriction such that $\mathcal{G}$ is total with respect to $\varrho$. Then $\mathcal{G}$ is total with respect to $\varrho \backslash Attr_\alpha(U)$ for all sub-mappings $U$ of $\varrho$ and each player $\alpha$.*

*Proof.* Let $\mathcal{G}$ and $\varrho$ be as stated. Consider an arbitrary mapping $U : V \to 2^{\mathfrak{C}}$, and let $A = Attr_\alpha(U)$ be the $\alpha$-attractor towards $U$. By Lemma 1, $A$ is a sub-mapping of $\varrho$. Towards a contradiction, assume that $\mathcal{G}$ is not total with respect to $\varrho \backslash A$. Then there is some vertex $v \in V$ and some configuration $c \in (\varrho \backslash A)(v)$ such that for all $w \in vE$, if $c \in \theta(v, w)$ then $c \notin (\varrho \backslash A)(w)$. Pick such a vertex $v$ and configuration $c$. Since $\mathcal{G}$ is total with respect to $\varrho$, we know that there is at least one $w \in vE$ with $c \in \theta(v, w)$ and $c \in \varrho(w)$. Let $w \in vE$ be such that $c \in \theta(v, w)$ and $c \in \varrho(w)$. It then follows that $c \notin (\varrho \backslash A)(w)$, and, hence, $c \in A(w)$. So, for all $w \in vE$ for which $c \in \theta(v, w)$ and $c \in \varrho(w)$ we have $c \in A(w)$. But then, by definition of $\alpha$-attractor, also $c \in A(v)$. Contradiction, since $c \in (\varrho \backslash A)(v)$. □

We proceed with the following result regarding the propagation of winning with respect to a sub-mapping along an attractor.

**Lemma 3.** *Let $\mathcal{G} = (V, E, \mathfrak{C}, p, \theta, (V_0, V_1))$ be a variability parity game and let $\varrho : V \to 2^{\mathfrak{C}}$ be a restriction. Let $\alpha$ be an arbitrary player and suppose $U$ is a sub-mapping of $\varrho$. If for all $v \in V$, player $\alpha$ wins vertex $v$ for all configurations $c \in U(v)$, then $\alpha$ wins vertex $v$ for all configurations $c \in Attr_\alpha(U)(v)$.*

*Proof.* Let $\varrho$, $\alpha$ and $U$ be as stated. We proceed by induction on $i$ with respect to the definition of $Attr^i_\alpha(U)$.

Base case ($i = 0$): Follows by assumption. Induction step ($i > 0$): Suppose player $\alpha$ wins vertex $v$ for all configurations $c \in Attr^i_\alpha(U)(v)$. Pick an arbitrary vertex $v'$ and configuration $c' \in Attr^{i+1}_\alpha(U)(v')$. Since $c' \in Attr^{i+1}_\alpha(U)(v')$, we have $c' \in \varrho(v)$. If $c' \in Attr^i_\alpha(U)(v')$, the result follows instantly by induction. If $c' \notin Attr^i_\alpha(U)(v')$, then we distinguish two cases.

Case $v' \in V_\alpha$: Then there must be some $w \in v'E$ such that $c' \in \theta(v', w)$ and $c' \in Attr^i_\alpha(U)(w)$. Let $w$ be such. Then player $\alpha$ can play a $c'$-configured token from $v'$ to $w$ and, by induction, win vertex $w$ for configuration $c'$. But then she also wins vertex $v'$ for configuration $c'$.

Case $v' \in V_{\bar{\alpha}}$. Then, for all $w \in v'E$ such that $c' \in \theta(v', w)$, also $c' \in Attr^i_\alpha(U)(w)$. Since regardless of how player $\bar{\alpha}$ moves the $c'$-configured token from $v'$ along an edge admitting $c'$, she will end up in a vertex that, by induction, is won by $\alpha$ for configuration $c'$. □

The next theorem captures the correctness of Algorithm 1.

**Theorem 2.** *Let $\mathcal{G} = (V, E, \mathfrak{C}, p, \theta, (V_0, V_1))$ be a variability parity game and let $\varrho : V \to 2^{\mathfrak{C}}$ be a restriction such that $\mathcal{G}$ is total with respect to $\varrho$. Then* Solve($\varrho$) *returns the mappings $W_0, W_1 : V \to 2^{\mathfrak{C}}$ such that for all $v \in V$, $W_0(v) \cup W_1(v) = \mathfrak{C}$ and both for player 0 and 1, for each $c \in W_\alpha(v)$, player $\alpha$ wins vertex $v$ for configuration $c$.*

*Proof.* Fix a total variability parity game $\mathcal{G} = (V, E, \mathfrak{C}, p, \theta, (V_0, V_1))$. We prove a slightly stronger property, *viz.* for all restrictions $\varrho : V \to 2^{\mathfrak{C}}$ such that $\mathcal{G}$ is total with respect to $\varrho$, procedure Solve($\varrho$) returns mappings $W_0, W_1 : V \to 2^{\mathfrak{C}}$ that are sub-mappings of $\varrho$ such that for all $v \in V$ it holds that $W_0(v) \cup W_1(v) = \varrho(v)$ and player $\alpha$ wins vertex $v$ for each configuration $c \in W_\alpha(v)$. Let us define $|\varrho| = \sum_{v \in V} |\varrho(v)|$. The proof will proceed by induction on $|\varrho|$ and closely follows the standard proofs of correctness for parity games.

Base case: We have $\varrho(v) = \emptyset$ for all $v \in V$. Consequently, the algorithm returns the functions $W_0$ and $W_1$ satisfying $W_0(v) = W_1(v) = \emptyset$ for all $v \in V$. Trivially $W_0$ and $W_1$ satisfy the statement.

Induction step: Let $\varrho$ be a restriction such that $\mathcal{G}$ is total with respect to $\varrho$. As our induction hypothesis, assume that the statement holds for all $\varrho'$ such that $|\varrho'| < |\varrho|$. Let $m$ be the maximal priority among those vertices in $\mathcal{G}$ for which $\varrho$ yields a non-empty set of configurations, and let $\alpha$ be $m \bmod 2$. Let $U$ be the sub-mapping of $\varrho$ for which $U(v) = \varrho(v)$ if $p(v) = m$, and $U(v) = \emptyset$ otherwise, and let $A$ be the sub-mapping $Attr_\alpha(U)$. By Lemma 2, $\mathcal{G}$ is total with respect to $\varrho \backslash A$, and hence, by induction, the functions $W'_0$, $W'_1$ returned by Solve($\varrho \backslash A$) satisfy the statement. Next, we distinguish two cases.

Case $W'_{\bar{\alpha}}(v) = \emptyset$ for all $v$. Then, by our induction hypothesis, player $\alpha$ wins all vertices $v$ for configurations $c \in W'_{\alpha}(v)$ in the game restricted to $\varrho \backslash A$. Regarding the remaining vertices, note that for vertices $v \in V_{\bar{\alpha}}$ and configurations $c \in W'_{\alpha}(v)$ with an edge to a vertex $w$ with $c \in A(w)$, player $\bar{\alpha}$ may escape to such vertices. However, then $\alpha$ can force the play to visit a vertex with priority $m$. Remaining in vertices with priority $m$ means losing for $\bar{\alpha}$. Playing to any vertex other than those in $U$ leads to a play that remains either in $W_{\alpha}$ or infinitely often revisits $U$. In either case, $\alpha$ wins such plays. For vertices $v \in V_{\alpha}$ and configurations $c \in \varrho(v)$, player $\alpha$ either follows the winning strategy in $W'_{\alpha}$ or the attractor strategy for $A$ towards a vertex in $U$. Consequently, $\alpha$ wins all vertices $v$ for all configurations $c \in \varrho(v)$, which is consistent with $W_{\alpha}$ and $W_{\bar{\alpha}}$ as returned by Solve.

Case $W'_{\bar{\alpha}}(v) \neq \emptyset$ for some $v$. Since player $\bar{\alpha}$ wins any vertex $v$ for configuration $c \in W'_{\bar{\alpha}}(v)$ in the game restricted to $\varrho \backslash A$, and player $\alpha$ cannot force the play to a vertex $w$ for which $c \in A(w)$, player $\bar{\alpha}$ also wins all such vertices and configurations in $\mathcal{G}$ restricted to $\varrho$. By Lemma 3, $\bar{\alpha}$ thus also wins all vertices $v$ for configurations $c \in B = Attr_{\bar{\alpha}}(W'_{\bar{\alpha}})(v)$. By Lemma 2, $\mathcal{G}$ is total with respect to $\varrho \backslash B$, and hence, by induction, the functions $W''_0, W''_1$ returned by the call Solve$(\varrho \backslash B)$ satisfy the statement. It then follows that player $\alpha$ wins all vertices $v$ for configurations $c \in W''_{\alpha}(v)$ and player $\bar{\alpha}$ wins all vertices $v$ for configurations $c \in (W_{\bar{\alpha}} \cup B)(v)$ as set by Solve. □

Algorithm 1 requires that the attractor $Attr_{\alpha}(U)$ for a sub-mapping $U$ can be computed (cf. line 8 of the algorithm). To cater for this, the attractor computation for sub-mappings can be implemented following the pseudo-code of Algorithm 2, the correctness of which is claimed by Lemma 4.

**Lemma 4.** *For a restriction $\varrho : V \to 2^{\mathfrak{C}}$, a sub-mapping $U : V \to 2^{\mathfrak{C}}$ of $\varrho$ and a player $\alpha$,* Attr$(\alpha, U)$ *terminates and returns a sub-mapping $A$ of $\varrho$ satisfying $A = Attr_{\alpha}(U)$.* □

Algorithm 2 is actually a straightforward implementation of the definition of the attractor set computation following the high-level structure of the attractor computation for standard parity games. We forego a detailed proof of Lemma 4, which, for soundness, uses an invariant stating that the computed sub-mapping $A$ under-approximates $Attr_{\alpha}(U)$ and for completeness uses an invariant that asserts for all configurations $c \in Attr_{\alpha}(U)(v)$ either $c \in A(v)$ or there is a vertex $v' \in Q$ and attractor strategy underlying $Attr_{\alpha}(U)(v)$ inducing a play for $c$, starting in $v$, visiting $v'$ and not visiting vertices $v''$ with $c \in A(v'')$ in between.

Instead, we briefly explain the underlying intuition. It conducts a typical backwards reachability analysis, maintaining a queue $Q$ of vertices that are at the frontier of the search for at least some configurations. For each vertex $w$ in this frontier, its predecessors $v \in Ew$ are inspected in a for-loop. Either such a predecessor is owned by player $\alpha$, in which case all configurations that can reach $w$ in one step are added to the attractor set for $v$, or such a predecessor is owned by player $\bar{\alpha}$, in which case *all* $v$'s successors must be inspected, and only those configurations $c$ of $v$ for which all their successor options are to move to some vertex $w'$ already satisfying $c \in A(w')$ are added to its attractor.

**Algorithm 2** Attractor computation. Given a variability parity game $\mathcal{G} = (V, E, \mathfrak{C}, p, \theta, (V_0, V_1))$, a restriction $\varrho : V \to 2^{\mathfrak{C}}$ and a sub-mapping $U$ of $\varrho$, the algorithm computes the $\alpha$-attractor towards $U$.

**function** $\text{ATTR}(\alpha, U)$
    Queue $Q \leftarrow \{ v \in V \mid U(v) \neq \emptyset \}$
    $A \leftarrow U$
    **while** $Q$ is not empty **do**
        $w \leftarrow Q$.pop()
        **for** every $v \in Ew$ such that $\varrho(v) \cap \theta(v, w) \cap A(w) \neq \emptyset$ **do**
            **if** $v \in V_\alpha$ **then**
                $a \leftarrow \varrho(v) \cap \theta(v, w) \cap A(w)$
            **else**
                $a \leftarrow \varrho(v)$
                **for** $w' \in vE$ such that $\varrho(v) \cap \theta(v, w') \cap \varrho(w') \neq \emptyset$ **do**
                    $a \leftarrow a \cap (\mathfrak{C} \setminus (\theta(v, w') \cap \varrho(w')) \cup A(w'))$
                **end for**
            **end if**
            **if** $a \setminus A(v) \neq \emptyset$ **then**
                $A(v) \leftarrow A(v) \cup a$
                **if** $v \notin Q$ **then** $Q$.push($v$)
            **end if**
        **end for**
    **end while**
    **return** $A$
**end function**

## 6 Implementation and Experiments

As an initial validation of our approach we experimented with two SPL examples, *viz.* the well-known minepump and elevator case studies first recognised as SPLs in [3, 14], modelled for the mCRL2 toolset [20, 21].

A prototype for solving variability parity games connecting to the mCRL2 toolset was implemented in `C++` using the BuDDy package [31, 32] for BDD operations. The prototype uses BDDs to represent product families; parity games are represented as graphs with adjacency lists for incoming and outgoing edges. For the recursive algorithm, bit vectors are used to represent sets of vertices sorted by parity then by priority. All experiments were run on a standard Linux desktop with Intel i5-4570 3.20Hz processor and 8GB DDR3 internal memory.[3]

### 6.1 Minepump Case Study

The minepump example of [33], in the SPL variant of [4], describes a configurable software system coordinating the sensors and actuators of a pump for mine drainage. The purpose of the system is to keep a mine shaft free from water.

[3] Solvers and experiments: https://github.com/SjefvanLoo/VariabilityParityGames

A controller operates a pump that may not start nor continue running in the presence of dangerously high levels of methane gas. To this end, it needs to communicate with sensors that measure the water and methane levels. The SPL model has 11 features and 128 products; the resulting FTS consists of 582 states and 1376 transitions. The mCRL2 code of this model, developed for [19], closely follows the fPROMELA code of [4] (also used in [16]) that is distributed with [8].

We verified nine properties, $\varphi_1$ to $\varphi_9$, for the minepump case study, examined also elsewhere in the SPL literature (cf., *e.g.* [3, 4, 7, 16, 19, 24, 34–36]). These induce variability parity games consisting of approximately 3000 to 9200 vertices and 2 to 4 different priorities. Specifically, for properties $\varphi_1$, $\varphi_4$, and $\varphi_7$, we used the following formulae, expressed in the mCRL2 variant of the modal $\mu$-calculus, which allows to mix fixed points, regular expressions, and first-order constructs.

*Property* $\varphi_1$*.* Absence of deadlock: `[true*] <true> true`

*Property* $\varphi_4$*.* The pump cannot be switched on infinitely often:

```
( mu X. nu Y. ([pumpStart] [!pumpStop*] [pumpStop] X &&
    [!pumpStart] Y )) && ( [true*] [pumpStart] mu Z. [!pumpStop] Z )
```

*Property* $\varphi_7$*.* The controller can always eventually receive/read a message, *i.e.* return to its initial state from any state: `[true*] <true*> <receiveMsg> true`

While $\varphi_4$ is a common LTL-type formula, $\varphi_7$ is typical for CTL. Table 3 provides the running times for verification of properties $\varphi_1$ to $\varphi_9$ via variability parity games, and the sizes of classes $(P^+, P^-)$ partitioning $\mathcal{P}$. The results show that the collective solving strategy for family-based SPL model checking outperforms the individual solving strategy for product-based SPL model checking.

While a full baseline comparison with other SPL model checking algorithms was not performed, our approach promises to be at least as efficient as related approaches. This conjecture is based on the running times reported for properties $\varphi_1$, $\varphi_4$, and $\varphi_6$ in [4, 16, 19] (all verified with standard computers of that time).

**Table 3.** Running times (**in ms**) for experiments for the product-based and family-based SPL model checking of the minepump and elevator case studies using recursive algorithm for variability parity games.

| Minepump SPL | | | | Elevator SPL | | | |
|---|---|---|---|---|---|---|---|
| Property | product | family | $\|P^+\| / \|P^-\|$ | Property | product | family | $\|P^+\| / \|P^-\|$ |
| $\varphi_1$ | 28.88 | 3.92 | 128/0 | $\psi_1$ | 14335 | 5409 | 2/30 |
| $\varphi_2$ | 54.79 | 6.76 | 0/128 | $\psi_2$ | 14988 | 5744 | 4/28 |
| $\varphi_3$ | 184.7 | 24.70 | 0/128 | $\psi_3$ | 16045 | 5020 | 4/28 |
| $\varphi_4$ | 145.0 | 37.46 | 96/32 | $\psi_4$ | 16865 | 5272 | 4/28 |
| $\varphi_5$ | 144.5 | 12.19 | 96/32 | $\psi_5$ | 8954 | 3013 | 16/16 |
| $\varphi_6$ | 242.9 | 42.79 | 112/16 | $\psi_6$ | 4252 | 772 | 32/0 |
| $\varphi_7$ | 134.3 | 11.71 | 128/0 | $\psi_7$ | 4171 | 765 | 32/0 |
| $\varphi_8$ | 17.44 | 1.058 | 128/0 | | | | |
| $\varphi_9$ | 110.0 | 6.853 | 0/128 | | | | |

#### 6.2 Elevator Case Study

The other configurable system we considered is the elevator example of [37] of a lift travelling between five floors. A product in the elevator system may or may not provide the features of parking, load and overload detection, cancelling on emptiness, and priority for specific floors. Absence or presence of specific features in a system configuration generally leads to different behaviour. The behaviour of the lift itself is governed by the so-called single button collective control strategy, deciding which floor is visited next. Roughly speaking, and dependent on the specific feature setting, the lift operates in sweeps, only changing direction if there are no outstanding calls in the current direction. The FTS implementation in mCRL2 underlying the experiments is derived from the 120 lines of SMV code presented in [37]. Although the number of features in this SPL example is small, *viz.* only 5 independent features resulting in 32 different configurations, the FTS consists of 95591 states and 622265 transitions.

The seven properties, $\psi_1$ to $\psi_7$ for the elevator case study, also examined elsewhere in the literature (cf., *e.g.* [10–12, 14, 15, 25, 26, 35, 38]), which we experimented with were adapted from [37]. These induce variability parity games consisting of approximately 440000 to 18500000 vertices with 2 to 3 different priorities. The properties cover a proper handling of requests, correct behaviour with respect to the control strategy, proper behaviour when idling, and the possibility to stop at floors while passing. By way of illustration, properties $\psi_2$, $\psi_3$, and $\psi_5$ are expressed as follows in the mCRL2 variant of the modal $\mu$-calculus.

*Property $\psi_2$.* Invariantly, if a lift button is pressed for a floor, the lift will eventually open its doors on this floor:

```
[true*] forall i:Floor. [liftButton(i)]
    ( mu X. ( [!open(i)] X && <true> true ) )
```

*Property $\psi_3$.* Invariantly, if the lift is travelling up while there are calls above the lift will not change direction:

```
[true*] ( ([ direction(up).
    (!(direction(down) || exists k:Floor. open(k)))* ]
        forall i:Floor. val(1 <= i && i <= 5) =>
            [ open(i) ] forall j:Floor. val(i < j && j <= 5) =>
                [ liftButton(j) ] mu Y. ( [!open(j)] Y &&
                    [direction(down)] false && < true > true ) ) )
```

*Property $\psi_5$.* Invariantly, if the lift is idling, it does not change floors:

```
( forall i:Floor. val(1 <= i && i <= 5) =>
    <true*.idling(i)> true ) &&
( [true*] forall i:Floor. val(1 <= i && i <= 5) =>
    [ idling(i) ] nu Y. <idling(i)> Y )
```

It is noted, in particular with regard to property $\psi_5$, that unlike the original SMV elevator system, our lift idles with its doors open, to prevent the situation where someone in the lift infinitely often presses the landing button for the current floor, keeping the process busy without the lift making any movement.

Also in the case of the elevator system we notice a significant difference in performance when doing product-based model checking calling the individual solving strategy or family-based model checking calling the collective solving strategy. The difference is, however, not that striking compared to the minepump case study, which, we believe, is due to the small number of different features.

As said, a full baseline comparison with other SPL model checking algorithms was not performed. For one, the efficiency of our approach with respect to related approaches is not easily measured with the elevator case study. While properties $\psi_2$ and $\psi_5$ were verified also in [14, 15, 25, 26, 35, 38], not much can be concluded from the reported running times. First, our model's mCRL2 code was developed from scratch, following the SMV code from [37], and not the fPROMELA code of [14, 15, 25, 26, 35, 38]. Moreover, the number of floors in these models ranges from 4 to 6. In [10–12], finally, the models are probabilistic, the number of floors ranges from 2 to 40, and different (probabilistic) properties were verified.

### 7 Conclusions

We have introduced variability parity games as a generalisation of parity games, reflecting the generalisation by FTSs of LTSs, and have defined the SPL model checking problem of modal $\mu$-calculus formulae on FTSs as a variability parity game solving problem, for which we have provided a recursive algorithm based on a collective, family-based solving strategy. To illustrate the efficiency of the approach, we have applied it to two classical examples from the SPL literature, *viz.* the minepump and the elevator case studies. The experiments show that the collective, family-based strategy of solving variability parity games typically outperforms the individual, product-based strategy of solving the standard parity games obtained by projection from the variability parity games

Further experiments are needed to measure and pinpoint the differences in efficiency. One direction for future work is to generate a sufficient number of random variability parity games to this aim. In particular, the configuration sets that label the edges of the variability parity games for the minepump and elevator case studies obey a very specific distribution, typically admitting either 100% or 50% of the configurations. It would be interesting to see how our approach behaves in case of SPLs with more complexly structured feature diagrams.

There is a wealth of different algorithms available for parity games, of which the recursive algorithm that we have here lifted to variability parity games is one of the most competitive ones in practice. Nevertheless, we think it pays to study other algorithms and lift these to variability parity games, too. Finally, we believe that variability parity games have applications beyond SPL model checking; *e.g.* in (parameter) synthesis problems. We leave these topics for future research.

*Acknowledgements* Work partially supported by the MIUR PRIN 2017FTXR7S project IT MaTTerS (Methods and Tools for Trustworthy Smart Systems).
